# Recovery from depressive symptoms, state anxiety and post-traumatic stress disorder in women exposed to physical and psychological, but not to psychological intimate partner violence alone: A longitudinal study

**DOI:** 10.1186/1471-244X-10-98

**Published:** 2010-11-25

**Authors:** Concepción Blasco-Ros, Segunda Sánchez-Lorente, Manuela Martinez

**Affiliations:** 1 Department of Psychobiology, Faculty of Psychology, University of Valencia, Spain

## Abstract

**Background:**

It is well established that intimate male partner violence (IPV) has a high impact on women's mental health. It is necessary to further investigate this impact longitudinally to assess the factors that contribute to its recovery or deterioration. The objective of this study was to assess the course of depressive, anxiety and post-traumatic stress disorder (PTSD) symptoms and suicidal behavior over a three-year follow-up in female victims of IPV.

**Methods:**

Women (n = 91) who participated in our previous cross-sectional study, and who had been either physically/psychologically (n = 33) or psychologically abused (n = 23) by their male partners, were evaluated three years later. A nonabused control group of women (n = 35) was included for comparison. Information about mental health status and lifestyle variables was obtained through face-to-face structured interviews.

**Results:**

Results of the follow-up study indicated that while women exposed to physical/psychological IPV recovered their mental health status with a significant decrease in depressive, anxiety and PTSD symptoms, no recovery occurred in women exposed to psychological IPV alone. The evolution of IPV was also different: while it continued across both time points in 65.21% of psychologically abused women, it continued in only 12.12% of physically/psychologically abused women while it was reduced to psychological IPV in 51.5%. Hierarchical multiple regression analyses indicated that cessation of physical IPV and perceived social support contributed to mental health recovery, while a high perception of lifetime events predicted the continuation of PTSD symptoms.

**Conclusion:**

This study shows that the pattern of mental health recovery depends on the type of IPV that the women had been exposed to. While those experiencing physical/psychological IPV have a higher likelihood of undergoing a cessation or reduction of IPV over time and, therefore, could recover, women exposed to psychological IPV alone have a high probability of continued exposure to the same type of IPV with a low possibility of recovery. Thus, women exposed to psychological IPV alone need more help to escape from IPV and to recuperate their mental health. Longitudinal studies are needed to improve knowledge of factors promoting or impeding health recovery to guide the formulation of policy at individual, social and criminal justice levels.

## Background

Intimate male partner violence (IPV) continues to be a major public health problem and has both short- and long-term mental health consequences for women, which result in a subsequent burden on the health care system and state [1-7]. This type of violence refers to actual or threatened physically, psychologically or sexually abusive acts committed against women by their current or former male partners. During the last three decades, cross-sectional, prospective and retrospective studies have consistently demonstrated that living with a violent intimate partner is a significant contributor to women's adverse mental health outcomes. The most prevalent sequelae include depression, anxiety and post-traumatic stress disorder (PTSD) [8-14]. Furthermore, IPV is strongly associated with suicidality, sleep and eating disorders, low self-esteem, personality disorders, social dysfunction and an increased likelihood of substance abuse [15-26].

Women exposed to IPV may experience different constellations of violence characterized by various combinations of physical, sexual and psychological violence. Although until recently, most research addressing the consequences of IPV on mental health focused on the impact of acts of physical violence, the concomitance with sexual violence has been reported to increase the negative effects [13,27,28], and the concomitance with psychological IPV *per se *is sufficient to predict mental health sequelae (12, 29-31). A high prevalence of all types of violence is associated with the highest prevalence of depression and PTSD [29,32]. On the other hand, the few studies that have assessed the influence of psychological IPV alone highlight the strong deterioration of mental health when compared to psychological IPV concomitant with physical IPV [13,14,33-36]. In summary, the results of previous research show that IPV is a complex experience of violence, and it is recommended that all types of IPV should be taken into account when assessing the association of IPV with women's mental health status.

Once studies have been performed to assess the incidence of mental health disorders in women experiencing IPV, it is necessary to determine what can be done to help them recover their health and quality of life. For this reason, longitudinal studies have been recommended by researchers to identify the changes in women's lives and the intervention programs that are beneficial or detrimental for recovery [37-42]. However, despite the growing awareness of this matter, few longitudinal studies have been carried out to date. In general, previous studies reported an improvement in mental health status over time with a decrease in depressive and anxiety symptoms as well as PTSD incidence [43-47]. The personal and social factors that have been reported to have beneficial effects on women's mental health recovery include the cessation of violence, the feelings of being safe and in control, the end of the relationship with the aggressive partner, the engagement of coping strategies and the existence of social support [46-52]. However, it has also been reported that mental health problems may persist long after the cessation of violence and that some women just out of the abusive relationship may have greater psychological difficulties than those who are still in it [43,46,53]. On the other hand, the factors that have been found to be detrimental for recovery are a lack of social support, greater severity and maintenance of the IPV, and an avoidant coping strategy [23,47,50-52].

In a previous cross-sectional study, we found that female victims of IPV had a higher incidence of depressive, anxiety and PTSD symptoms and also had a higher incidence of suicidal thoughts and attempts than women not exposed to IPV. There were no differences between women exposed to physical and psychological IPV and those exposed to psychological IPV alone [13]. Consequently, the main aim of the current study was to explore the course of mental health status over a follow-up period of three years in the women that participated in the previous study. The second objective was to determine the factors that contributed to either the recovery or the deterioration of women's mental health by focusing on sociodemographic variables, medical treatment, evolution of the IPV and the relationship with the aggressor, a lifetime history of victimization, and the perception of life events and social support.

## Methods

### Participants

The present study is part of a larger longitudinal research project in which women who had participated in a previous cross-sectional study conducted between 2000-2002 on the impact of IPV on health (T-1: baseline) [13,21,54-56] were evaluated again three years later (T-2). These women, who had been either physically/psychologically (n = 33) or psychologically abused (n = 23) by their male partners, had originally been recruited through the Centers for Helping Women (which offers information, help from lawyers and social workers, and some psychological interventions for the women) in the three provinces of the Valencian Community of Spain (Alicante, Castellon and Valencia). A control group of women (n = 35) not exposed to IPV was recruited for the project through women's clubs and was included for comparison. For the follow-up assessment, all of the women were contacted again by phone and invited to participate. The study was approved by the University of Valencia research ethics committee, and, after the study was completely described to the subjects, written informed consent was obtained. Subjects did not receive any money or other incentive for their participation. All participants were of Spanish nationality.

### Design

The second assessment of the wider study consisted of a structured interview during which two trained female psychologists asked women about their life and health status during the three-year follow-up period. Interviews took place either in the Centers or in the women's homes if conditions were sufficiently safe to allow it. In general, each woman was interviewed by the same psychologist 4-6 times due to the length of the questionnaires, with each session taking 1.5 hours. The results presented in this paper correspond to the course of recovery of mental health status.

A comprehensive questionnaire was designed for a face-to-face structured interview. Most of the questions were devised to yield objective factual reports. All questionnaires were administered at both T-1 and T-2 except the childhood abuse questionnaire (only at T-1) and the life events and social support questionnaires (only at T-2). The questionnaires from which information for the present study was obtained are described below.

### Questionnaires

1)-Sociodemographic profile included age and education level.

2)-Intervention treatment included psychological and psychiatric treatment as well as psychopharmacological (antidepressants, anxiolytics and hypnotics) treatment that women had received.

3)-Evolution of the relationship with the aggressor/partner

Detailed information about the nature of the relationship between the woman and the aggressor/partner (marital status and cohabitation) was acquired.

4)-Evolution of intimate partner violence

Detailed information about the pattern of IPV over time was obtained. A questionnaire was constructed to collect specific data about the different types of violence (physical, psychological and sexual) perpetrated by the abusive partner. Each type consisted of one or more of the acts described below. Women were asked to answer "yes" or "no" to the experiencing of each act.

*Psychological violence* included verbal attacks (insults, humiliations); control and power (isolation from family and friends, impeded decision-making, economic abandonment); pursuit and harassment, verbal threats (threats on the life of the woman or her family, threats regarding custody of children, intimidating phone calls); and blackmail (economic or emotional).

*Physical violence* included punches, slaps, kicks, pushes, bites and strangling.

*Sexual violence* included forced sex (vaginal or anal penetration, oral sex from her to him or from him to her, objects inserted in vagina or anus) and forced or coerced use of pornographic films and photos.

The detailed information given in this paper refers to the previous violent male partner from T-1. Control women were also asked all of the same questions to ensure that they had not experienced IPV at any time. Confirmation or not of any of the acts of physical, psychological or sexual violence was used as the criterion to designate women as abused or nonabused. The occurrence of any acts of physical violence was used to classify abused women into two groups: physically/psychologically abused or psychologically abused.

5)-Lifetime history of victimization

In the previous T-1 study, information about the experience of abuse independent of the IPV (both during childhood and adulthood) was obtained [see 55 for detailed information]. In the present follow-up study, information was also acquired about any violence perpetrated by individuals other than the previous partner during the interval leading up to T-2.

6)-Functional social support

The Duke-UNC scale (11 item version) was used to measure functional social support [57]. The questionnaire includes 11 Likert-type items with 5 answer options scored from 1 to 5 (ranging from "much less than desired" to "as much as desired"). It has two dimensions, i.e., confidential and affective, and a cut-off point to classify perceived social support as low (≤ 32) or normal (> 32). The Spanish version of this questionnaire was validated in Spain by Bellón et al. (1996) [58]. The internal consistency of the scale and subscales (confidential and affective) were 0.90, 0.88, and 0.79, respectively. The reliability of the administration of the scale by an interviewer was 0.80 (for the Spanish validation).

7)-Life events

A questionnaire was designed by the research team with the main objective of gathering information about life events (total number and type) that were spontaneously identified by the women as relevant during the interval between T-1 and T-2. Women could speak freely about as many events as desired or none. Additionally, the degree to which these events forced women to readjust their lives was determined. For this rating, the women were asked to give a subjective weight to each event using a continuous scale from 1 to 10 (1 was the best event and 10 the worst). A total score given by women for each type of event was calculated.

### Mental health assessment

#### 1)-Depressive symptoms

The severity of depressive symptoms was measured with the Beck Depression Inventory (BDI) [59]. Total scores of the BDI ranged from 0 to 63. The Spanish version of BDI used in this study was validated by Conde and Useros (1975) [60], who obtained a coefficient of internal consistency of 0.88. Several studies support the internal consistency and construct validity of this Spanish version [61,62]. The Cronbach's alpha coefficient of the BDI scale was 0.90. In this study, the cut-off score was set at 18.

#### 2)-State anxiety

Spielberger's State-Trait Anxiety Inventory (STAI) was used to measure levels of state anxiety symptoms [63]. The present study employed the Spanish version of the STAI, which was validated and adapted by TEA Editions (1988) [64].

#### 3)-Post-traumatic stress disorder

The incidence and severity of symptoms of current PTSD were assessed with Echeburua's Severity of Symptom Scale of Post-traumatic Stress Disorder [65]. This scale is a structured interview based on DSM-IV criteria [66]. The instrument has a high internal consistency, with a Cronbach's alpha coefficient of 0.92 and a high test-retest reliability, as well as good discriminant, concurrent and construct validity. The Criterion A stressor was assessed by asking the woman whether she had experienced an unusual, extremely distressful event (irrespective of whether it was IPV-related or not). Either type of event was considered a qualifying trauma when it met the DSM-IV criteria for PTSD and when symptoms of distress persisted for at least 4 weeks.

#### 4)-Thoughts and attempts of suicide

Women were asked about their lifetime incidence of thoughts and attempts of suicide at T-1 and during the follow-up period.

### Data analysis

Women were classified into three groups, i.e., nonabused, psychologically abused and physically/psychologically abused, depending on the type of IPV suffered at T-1. The three groups were compared with respect to age, perceived social support and lifetime events, and profile of mental health status using one-way analysis of variance (ANOVA). Level of education, marital status and cohabitation with the aggressor/partner, prevalence of childhood abuse, witnessing violence between parents during childhood, and adulthood victimization by individuals other than the partner were compared using Pearson χ^2 ^tests. To compare the mental health measures (depressive symptomatology, anxiety, and PTSD) over time, repeated-measures ANOVAs were performed with the factors of Time and Group to test the temporal effect. Post hoc comparisons were conducted with the Dunnett's T3 test. Student's *t*-test and McNemar's test were used for within-group comparisons in each group. To determine the relationship between mental health recovery (the difference between T-1 and T-2 scores in depressive, anxiety, and PTSD symptoms) and the course of IPV from T-1 to T-2, lifetime victimization, social support and the other sources of stress, hierarchical multiple regression analyses were carried out after controlling for age, education, psychopharmacological treatment, and mental health status at T-1. B coefficients, estimated odds ratios (ExpB) for each independent variable in the model, and the confidence intervals for the estimated odds ratios were calculated. The level of significance for all analyses was set at 0.05. All the analyses were conducted using SPSS version 16 and PASW version 17.

## Results

### Characteristics of the participants

A sample of 91 women participated in the follow-up study (T-2). They were categorized into three groups depending on the type of IPV suffered at T-1: nonabused (n = 35), psychologically abused (n = 23) and physically/psychologically abused (n = 33) women. There were no differences between groups at T-2 in terms of age [Vw(2,53.97) = 0.11; p = 0.89] or education level (Fisher; p = 0.74) (Table 1).

**Table 1:** Characteristics of nonabused, psychologically abused and physically/psychologically abused women (%)

	Nonabused		Psychologically		Physically/Psychologically	
Variable	women		abused women		abused women	
	(n = 35)		(n = 23)		(n = 33)	
	Time 1	Time 2	Time 1	Time 2	Time 1	Time 2
**Age**	45.14 ± 12.82	47.91 ± 12.8	45.6 ± 10.22	48.61 ± 10.06	44.93 ± 10.81	47.89 ± 10.72
**Education level**						
Able to read and write	2.9	2.9	4.3	4.3	9.1	6.1
Incomplete primary school	17.1	17.1	17.4	17.4	18.2	18.2
Primary school	40	40	52.2	52.2	39.4	42.4
Secondary school	28.6	28.6	26.1	26.1	21.2	21.2
University studies: 3-4 years	5.7	5.7	0	0	0	0
University studies: 5-6 years	5.7	5.7	0	0	12.1	12.1
**Marital status with aggressor/partner**						
Married	82.9	85.7	52.2	26.1	30.3	15.2
Single not living with partner	0	2.9	13	17.4	21.2	18.2
Separated/divorced	0	0	21.7	47.8	42.4	54.5
Single living with partner	14.3	8.6	13.0	8.7	6.1	12.1
Widow	2.9	2.9	0	0	0	0
**Cohabitation with the aggressor/partner** ** at the time of the interviews**	97.1	94.3	69.6	34.8	45. 5	27.3
**Intervention treatment**						
Psychiatric/psychological	28.6	8.6	56.5	26.1	60.6	50
Psychopharmacological	25.7	25.7	26.1	34.8	51.5	33.3
**Lifetime history of victimization**						
Childhood abuse	54.3	-	69.6	-	63.6	-
Physical	34.3	-	56.5	-	45.5	-
Psychological	31.4	-	34.8	-	45.2	-
Sexual	14.3	-	34.8	-	55.2	-
Childhood witnessing of violence between parents	17.1	-	21.7	-	36.4	-
Adulthood abuse by individuals other than partners	51.4	8.6	52.2	17.4	63.6	51.5
Physical	14.3	0	13.0	0	33.3	12.1
Psychological	34.3	5.7	30.4	17.4	50.0	42.4
Sexual	17.1	2.9	39.1	0	24.2	6.1
**Intimate partner violence**						
Physical	0	0	0	0	100	27.3
Psychological	0	0	100	82.6	100	78.8
Sexual	0	0	8.7	8.7	33.3	6.1
**Perceived social support: DUKE-UNC-11**	-	46.24± 6.92	-	40.3 ± 10.89	-	38.12 ± 12.97**
**Perception of total lifetime events**	-	13.94 ± 9.16	-	12.77 ± 9.8	-	18.06 ± 10.73

**: Differs from nonabused group at the same time point: p < 0.01

-: Indicates no incidence (% = 0)

### Course of relationship and cohabitation with the aggressor/partner

There was a significant association between IPV and marital status both at T-1 and T-2 (Fisher; p < 0.0005) (Table 1). The percentage of "married" women was higher than expected by chance in the nonabused group of women at both time points and was lower than expected at T-1 in the physically/psychologically abused women and at T-2 in both abused groups. The opposite pattern was observed in the category of "separated/divorced" women. On the other hand, there was a significant association between IPV and cohabitation with the aggressor/partner at the time of the interviews at both T-1 [χ^2^(2, N = 91) = 22.29; p < 0.0005] and T-2 [χ^2^(2, N = 91) = 35.87; p < 0.0005]. At both time points, the percentage of women cohabiting with the aggressor/partner was significantly higher and lower than expected by chance in the nonabused and physically/psychologically abused women, respectively. Additionally, the percentage was significantly lower than expected in psychologically abused women at T-2. On the other hand, the percentage of women cohabiting with the aggressor was associated with time in the psychologically abused group (McNemar; p = 0.008), with a decrease over the follow-up period.

### Evolution of intimate partner violence

**Figure 1:**
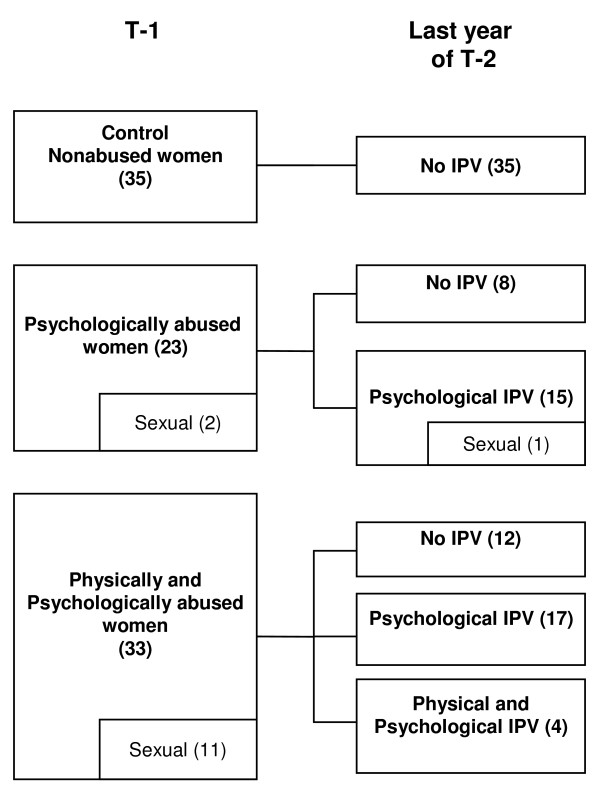
**Evolution of intimate partner violence**. Women were categorized into three groups depending on the type of IPV (intimate partner violence) suffered at T-1 (Time 1, baseline). The type of IPV that women were exposed to changed over the three year follow-up period (T-2). The concomitance of sexual IPV is included in each category.

The type of IPV that women were exposed to changed over the follow-up period (Figure 1). During the year prior to T-2, IPV ceased in 34.8% but continued in 65.2% of the women who were psychologically abused at T-1. Concerning the evolution of sexual IPV, only one woman continued to be exposed to it concomitantly with psychological IPV. Of the women who were physically and psychologically abused at T-1, IPV completely ceased in 36.4%, was reduced to psychological IPV alone in 51.5%, and continued as both physical and psychological IPV in 12.1%. None of the nonabused women experienced IPV during the follow-up period.

### Lifetime history of victimization

There was a history of childhood abuse and childhood witnessing of violence between parents in all three groups (Table 1), with no association with adult experiences of IPV (childhood abuse: [χ^2^(2, N = 91) = 1.47; p = 0.48]; childhood witnessing: [χ^2^(2, N = 91) = 3.53; p = 0.17]). On the other hand, there was a significant association between the violence perpetrated by people other than the intimate partner during the follow-up period (T-2) and IPV [χ^2^(2, N = 91) = 17.41; p < 0.0005]. The incidence was higher than expected by chance in the physically/psychologically abused group and lower in the nonabused group.

### Social support

There were significant differences between groups in the perception of social support as measured by the Duke-UNC-11 during the follow-up period (T-2) [Vw(2;47.84) = 6.36; p = 0.004]. Post hoc comparisons revealed a lower level of perceived social support in the physically/psychologically abused women in comparison to the nonabused women (p = 0.008). Differences were found in the confidant [Vw(2;47.82) = 6.18; p = 0.004] and in the affective scale [Vw(2;48.9) = 4.59; p = 0.015].

### Lifetime events

There were no differences between groups in the subjective perception of lifetime events experienced during the follow-up period [F(2,85) = 2.24; p = 0.11].

### Other control variables

There was an association between the percentage of women receiving psychiatric/psychological treatment and IPV at T-1 [χ^2^(2, N = 91) = 8.05; p = 0.02] and at T-2 [χ^2^(2, N = 90) = 14.35; p = 0.001]; the incidence was lower than expected in the nonabused group and higher in the physically/psychologically abused group (Table 1). Psychopharmacological treatment was significantly associated with IPV at T-1 [χ^2^(2, N = 91) = 6.07; p = 0.054]; it was more frequent than expected in the physically/psychologically abused group, with no association at T-2 [χ^2^(2, N = 91) = 0.07; p = 0.74].

### Course of recovery of mental health

Detailed information about the course of depressive, anxiety and PTSD symptoms as well as the incidence of thoughts and attempts of suicide is given in Table 2.

**Table 2:** Depression, anxiety, PTSD, and suicidal behavior in nonabused, psychologically abused, and physically/psychologically abused women

	Nonabused		Psychologically		Physically/Psychologically	
	Women		abused women		abused women	
	(n = 35)		(n = 23)		(n = 33)	
Variable	Time 1	Time 2	Time 1	Time 2	Time 1	Time 2
**BDI**	5.97 ± 5.51	6.31 ± 6.99	14.13 ± 8.78***	13.61 ± 11.01*	15.67 ± 12.65***	11.21 ± 12.00^##^
**State anxiety (STAI)**	10.06 ± 6.12	13.20 ± 10.87	20.74 ± 14.87**	19.09 ± 13.71	25.36 ± 14.92***	18.76 ± 12.45^#^
**PTSD**						
Incidence	0	2.9	39.1	21.7	24.2	18.2
Total score	1.82 ± 3.02	1.89 ± 5.90	13.91 ± 10.94***	10.09 ± 14.22*	14.55 ± 12.16***	7.00 ± 11.64^##^
Subscales PTSD score						
Re-experiencing	0.89 ± 1.23	0.69 ± 1.94	4.91 ± 3.94***	3.91 ± 5.23*	4.67 ± 3.97***	2.30 ± 3.41^##^
Avoidance	0.51 ± 1.20	0.60 ± 2.45	4.87 ± 4.21***	3.17 ± 5.43	5.18 ± 5.47***	2.39 ± 4.71^##^
Arousal	0.43 ± 0.95	0.60 ± 2.13	4.13 ± 4.19***	3.00 ± 4.33^+^	4.70 ± 3.84***	2.30 ± 4.07^##^
**Suicidal thoughts**	8.6	2.9	39.1	13^#^	60.6	6.5^###^
**Suicidal attempts**	2.9	0	17.4	0	33.3	0

BDI, Beck's Depression Inventory

STAI, Spielberger's State-Trait Anxiety Inventory

PTSD, post-traumatic stress disorder

*: Differs from nonabused group at the same time point, p < 0.05; **: p < 0.01; ***: p < 0.001

^#^: Differs from Time 1 in the same group, p < 0.05; ^##^: p < 0.01; ^###^: p < 0.001

^ +^: Differs from nonabused group at the same time point, p = 0.057

#### Depressive symptoms

There was a significant Group by Time interaction effect (MANOVA) in the score of self-rated depressive symptoms [F(2,88) = 3.18; p = 0.047]. The differences between groups were higher at T-1 [V_w_(2,46.56) = 13.50; p < 0.0005] than at T-2 [V_w_(2,48.09) = 4.96; p = 0.011]. Post-hoc comparisons indicated that while at T-1 both abused groups had more severe depressive symptoms than the nonabused group (p = 0.001), at T-2 only the psychologically abused group continued to have higher levels than the nonabused group (p = 0.023). Within-group comparisons over time (from T-1 to T-2) indicated that the physically/psychologically abused women showed a statistically significant decrease in depressive symptoms [t(32) = 2.93; p = 0.006], whereas the other two groups had not changed significantly (psychologically abused: [t(22) = 0.28; p = 0.78]; nonabused [t(34) = -0.29; p = 0.77]).

#### State anxiety

There was a significant Group by Time interaction effect (MANOVA) for reported state anxiety [F(2,88) = 4.49; p = 0.014]. There were differences between groups at T-1 [(V_w_(2,42.26) = 18.20; p < 0.0005] but not at T-2 [F(2,88) = 2.35; p = 0.10]. Post hoc comparisons indicated that at T-1, both abused groups had more severe anxiety symptoms than the nonabused group (psychologically abused: p = 0.009; physically/psychologically abused group: p < 0.0005). Within-group comparisons indicated a decrease in state anxiety in the physically/psychologically abused group [t(32) = 2.39; p = 0.023], whereas there was no significant change in the other two groups (nonabused group: [t(34) = -1.58; p = 0.12]; psychologically abused group: [t(22) = 0.67; p = 0.51]).

#### PTSD

The percentage of women that met the full diagnostic criteria for PTSD was associated with IPV at both T-1 ([χ^2^(2, N = 91) = 15.04; p = 0.001] and T-2 (Fisher; p = 0.042). At T-1, this percentage was higher than expected in the psychologically abused group and lower in the nonabused group. At T-2, it was only lower than expected in the nonabused group. The incidence of PTSD was not associated with Time in either of the two abused groups (psychologically abused group: McNemar p = 0.14; physically/psychologically abused group: McNemar p = 0.36). On the other hand, there was a significant Group by Time interaction effect (MANOVA) [F(2,88) = 3.86; p = 0.025] and a Time effect [F(1,88) = 9.84; p = 0.002] for the total score of PTSD. There were significant differences between groups at both T-1 [V_w_(2,38.23) = 28.45; p < 0.0005] and T-2 [V_w _(2,43.50) = 5.19; p = 0.01]. Post hoc comparisons indicated that at T-1, both abused groups had higher total PTSD scores than the nonabused group (p < 0.0005), while at T-2 only the psychologically abused group had higher scores than the nonabused group (p = 0.041). Within-group comparisons indicated a decrease in PTSD symptoms in the physically/psychologically abused group [t(32) = 3.31; p = 0.002], but there was no significant change in the other two groups (nonabused group: [t(34) = -0.05; p = 0.96]; psychologically abused group: [t(22) = 1.32; p = 0.20]). Detailed information about the subscales of re-experiencing, avoidance and arousal is given in Table 2. Statistical differences for the subscale scores were similar to those observed for the total score.

#### Thoughts and attempts of suicide

Thoughts and attempts of suicide were associated with IPV only at T-1 (thoughts: [χ^2^(2, N = 91) = 20.38; p < 0.0005]; attempts: [χ^2^(2, N = 91) = 10.89; p = 0.004]). Both of these incidences were higher than expected in the physically/psychologically abused and lower in the nonabused group. The percentage of women that had suicidal thoughts was associated with Time in both abused groups (physically/psychologically abused: [McNemar; p < 0.0005]; psychologically abused [McNemar; p = 0.03]) but not in the nonabused group: (McNemar; p = 0.25). Because there were no suicide attempts at T-2 in either group, no statistical analysis related to the change over the follow-up period was possible.

### Variables contributing to the recovery of mental health

To determine the variables that contributed to the recovery from depression, anxiety and PTSD symptomatology for the physically/psychologically abused women, hierarchical multiple regression analyses were conducted (Table 3). The analyses showed that with regard to the change in depressive symptoms over time, overall control variables (age, psychopharmacological treatment and depressive baseline scores) were significant predictors over the three-year follow-up period [ΔR^2 ^= 0.31, *F *= (3,83) = 12.22, R^2 ^= 0.31; p = 0.001]. Psychopharmacological treatment at T-2 (β = -0.44, p = 0.001) and depressive symptoms at T-1 (β = -0.62, p = 0.001) were the primary factors; less psychotropic drug use during the follow-up period and more depressive symptoms at T-1 predicted higher recovery. On the other hand, the overall variables of stress at T-2 were also significant predictors of the change over time [ΔR^2 ^= 0.11, *F *= (3,76) = 5.17, R^2 ^= 0.44; p = 0.003]. Perceived social support at T-2 (β = 0.40, p = 0.001) was the primary factor, and higher social support during the follow-up predicted greater recovery. Additionally, although the evolution of IPV from T-1 to the previous year of T-2 did not account for the change over time, cessation of physical IPV had an independent significant effect (β = 0.23, p = 0.05), and a more marked reduction in physical violence predicted a higher recovery.

**Table 3:** Hierarchical regression analyses for depression, anxiety, and PTSD recovery in nonabused, psychologically, and physically/psychologically abused women

	Depression					Anxiety					PTSD				
**Step and predictors**	**Total R**^**2**^	**R**^**2 **^**change**	**F change**	**β**	**t**	**Total R**^**2**^	**R**^**2 **^**change**	**F change**	**β**	**t**	**Total R**^**2**^	**R**^**2 **^**change**	**F** **change**	**β**	**t**

**Step 1**															
**Control variables**	0.31	0.31	12.22***			0.41	0.41	19.33***			0.29	0.29	11.24***		
Age T2				0.09	-0.91				0.07	0.78				0.01	0.12
Psychopharmacological treatment T2				-0.44	-4.53***				-0.20	-2.21*				-0.21	-2.15*
Scores at T1															
Depressive symptomatology				0.62	5.43***										
State Anxiety									0.73	6.82***					
PTSD														0.56	4.81***
Step 2															
**Lifetime history of victimization**	0.33	0.02	0.67			0.42	0.01	0.21			0.29	0.006	0.17		
Childhood abuse				0.09	0.89				0.09	0.91				0.03	0.27
Violence witnessed between parents				0.04	0.37				0.03	0.35				0.07	0.67
Adulthood violence by other than partners T1				-0.22	-0.23				0.09	0.99				0.08	0.81
Adulthood violence by other than partners T2				0.07	0.72				0.01	0.01				0.01	0.08
*Step 3*															
**Variables of stress at T2**	0.44	0.11	5.17**			0.50	0.07	3.43**			0.37	0.08	3.15*		
Cohabitation with the aggressor				-0.07	-0.49				0.11	0.79				-0.15	-0.99
Perception of lifetime events				-0.02	-0.25				-0.16	-1.69				-0.21	-2.03*
Perceived social support				0.4	3.54***				0.37	3.39***				0.24	2.13*
*Step 4*															
**Evolution of IPV from T1 to the previous year of T2**	0.47	0.03	0.87			0.51	0.02	0.57			0.43	0.06	1.52		
Continuation of physical IPV				0.07	0.70				0.42	0.67				-0.07	-0.65
Cessation of physical IPV				0.23	1.95*				0.12	0.55				0.27	2.22*
Continuation of psychological IPV				-0.09	-0.63				0.08	0.55				-0.13	-0.84
Cessation of psychological IPV				-0.12	-1.06				-0.02	-0.12				-0.23	-1.29
Cessation of sexual IPV				-0.04	-0.36				0.05	0.45				-0.09	-0.81

β = Standardized regression coefficient; IPV = intimate partner violence; T1 = Time 1; T2 = Time 2

*p < 0.05; ** p < 0.01; *** p < 0.001

Similarly, overall control variables [ΔR^2 ^= 0.41, *F *= (3,83) = 19.33, R^2 ^= 0.41; p = 0.001] and overall variables of stress at T-2 [ΔR^2 ^= 0.073, *F *= (3,76) = 3.63, R^2 ^= 0.49; p = 0.017] were significant predictors of the change in anxiety symptoms over time, with psychotropic drug use at T-2 (β = -0.20, p = 0.03), anxiety baseline scores at T-1 (β = 0.73, p = 0.001) and perceived social support at T-2 (β = 0.37, p = 0.001) as the primary factors. In contrast, cessation of physical IPV was not a significant predictor of recovery from anxiety. Finally, the variables that contributed significantly to the change in PTSD symptoms over time were similar to those for depression and anxiety; these variables included the overall control variables [ΔR^2 ^= 0.29, *F *= (3,83) = 11.24, R^2 ^= 0.29; p = 0.001], with the psychotropic drug use at T-2 (β = -0.21, p = 0.035) and the PTSD baseline scores at T-1 (β = 0.56, p = 0.001) as the primary factors, and the overall variables of stress at T-2 [ΔR^2 ^= 0.08, *F *= (3.76) = 3.15, R^2 ^= 0.37; p = 0.03], with the perception of lifetime events (β = -0.21, p = 0.046) and social support (β = 0.24, p = 0.036) as the primary factors. A higher perception of lifetime events predicted less recovery, and higher social support predicted greater recovery. Additionally, cessation of physical IPV was a significant independent predictor of recovery (β = 0.27, p = 0.030).

## Discussion

### Impact of IPV on mental health

This study examined the mental health status in women who had been exposed to psychological IPV alone or concomitant physical and psychological IPV longitudinally over a follow-up period of three years. The initial assessment indicated that both groups of abused women had more severe depressive, anxiety and PTSD symptomatology as well as a higher incidence of thoughts and attempts of suicide than nonabused control women. These results indicate that psychological IPV both alone and concomitant with physical IPV have similar consequences on women's mental health, as we and other researchers have previously reported [13,14,33-35]. Because of the impact of IPV on mental health, a high percentage of women exposed to this type of violence received psychiatric and psychological intervention, and half of those exposed to physical and psychological IPV used psychotropic drugs.

### Different courses of recovery

The main finding of the present study was that women exposed to concomitant physical and psychological IPV (physically/psychologically abused group) underwent a recovery of their mental health status with a significant decrease in depressive, anxiety and PTSD symptomatology over the 3-year follow-up period in comparison with the initial assessment. Consequently, differences between these women and those who were nonabused no longer existed. This course of recovery agrees with previous studies in which a decrease in depression and anxiety as well as the incidence of PTSD was reported over time for women exposed to IPV [41,43,44,46,47,50]. These findings are very important as they give hope to women whose mental health has deteriorated because of being victims of physical and psychological IPV. On the contrary, in the present study no recovery was found in women who had been exposed to psychological IPV alone (psychologically abused group), as they continued to have higher levels of depressive and PTSD symptomatology than nonabused women after the follow-up period of three years, with no significant decrease over time in any of the three assessed mental disorders. However, the incidence of suicidal thoughts and attempts was reduced over time in both abused groups, which might be because the period of time referred to for T-1 was the span of the entire lifetime, whereas at T-2 the period of time only referred to the three-year follow-up period.

### Factors contributing to the course of recovery

Thus, the present results indicate a different course of mental health status between women who had been exposed to psychological IPV alone and those who had suffered both physical and psychological IPV. It is therefore important to determine which personal and social factors contributed to this different pattern and, more specifically, which factors contributed to recovery or to the continuation of the compromised mental health. To this end, hierarchical multiple regression analyses were carried out that showed that the baseline score for each disorder at the initial assessment was a predictor of recovery. This finding indicates that a high level of deterioration did not impede improvement. On the other hand, perceived social support contributed to recovery for the three mental disorders, and the cessation of physical IPV contributed to recovery for depressive and PTSD symptoms. On the contrary, a high intake of psychotropic drugs predicted the continuation of the three disorders, and a high perception of lifetime events contributed to the continuation of PTSD symptoms. Thus, of all variables studied, the only one that impeded the recovery over time was the perception of lifetime events with respect to PTSD symptoms.

The evolution of IPV was different in women exposed to psychological IPV alone when compared to those experiencing both physical and psychological IPV. While IPV continued across both time points in 65.21% of women suffering psychological IPV alone, it continued in only 12.12% of women exposed to physical and psychological IPV and was reduced to psychological IPV alone in 51.5% of participants. This finding may explain why there was a more notable improvement in the women exposed to physical and psychological IPV compared to those experiencing psychological IPV alone. The regression analysis showed that the cessation of physical IPV contributed to the recovery of depressive and PTSD symptoms, which agrees with previous studies [44,46]. Thus, while women experiencing physical IPV have a higher likelihood of undergoing a cessation or reduction of IPV over time, women exposed to psychological IPV alone have a high probability of continued exposure to the same type of IPV. Factors contributing to these differences deserve increased attention. Thus, understanding the factors that contribute to women's responses to IPV that allow them to become free of the violence is of relevant importance. Some studies have started to assess this issue [33,67,68], as it has an important impact on the pattern of recovery of women's mental health. Another relevant aspect to take into account is that in most cases, psychological IPV did not cease while women were cohabiting with the aggressor, and it continued even when the women had separated from the aggressor [43] (authors' unpublished data).

The present results reveal that a high level of perceived social support was a significant predictor of recovery from the three mental disorders over the follow-up period. This finding agrees with the literature, which shows that social support protects against the effects of IPV on mental health and has a beneficial effect on women's decision to take actions to eliminate IPV, thus providing a beneficial impact on their health [9,44,49,67,69-72]. Furthermore, social support decreases the risk of revictimization by partners [49,73]. Thus, all studies highlight the buffering effects of social support on the impact of IPV on women's mental health and the beneficial effects of social support for recovery over time. On the other hand, the finding that a low-level use of psychotropic medication during the follow-up was a predicting factor of recovery from the three mental disorders indicates that women with more deteriorated mental health status are those who have a higher intake. Previous studies and our own study demonstrate that female victims of IPV take more psychoactive drugs than nonabused women [13,74].

Our results demonstrate that psychological IPV alone is not only highly detrimental to women's mental health but also reduces the likelihood of mental health recovery. These results are important, as psychological IPV is often still considered a minor type of violence and consequently receives less attention than physical IPV by clinicians, lawyers, policy makers, researchers and the female victims themselves. Thus, exposure to psychological IPV alone can no longer be considered a minor type of IPV when assessing and recognizing the impact of IPV on women's mental health. More importantly, psychological IPV alone is more resistant to cessation than physical IPV or psychological IPV concomitant with physical IPV. The possibility of exposure to psychological IPV alone should be considered in patients who have persistent mental problems.

## Strengths and limitations

The design of this investigation has a number of noteworthy strengths including its longitudinal design and the wide assessment of mental health that allowed us to study depression, anxiety, PTSD and suicidality. However, limitations include the sample size and the fact that the female participants were recruited from the Centers for Helping Women. Studies need to be carried out with different samples of women recruited from different settings. Population-based studies would be helpful to assess whether the pattern of mental recovery and the contributing factors to it identified here are generally applicable. The short follow-up time is another limitation of the study.

## Conclusions

These findings clearly indicate that the recovery of mental health is possible in women whose mental health has deteriorated because of being exposed to IPV. However, special emphasis must be placed on the fact that while being exposed to physical IPV is a predictor for the recovery of mental health over time, women exposed to psychological IPV alone need more help to escape from IPV and to recover their mental health status. Thus, further studies following the course of women's mental health are urgently required. Finally, the recovery of health by women exposed to IPV deserves the full attention of researchers, clinicians, lawyers and policy makers. Improved knowledge of outcomes, together with an understanding of factors promoting or impeding recovery, should guide the formulation of policy at individual, social and criminal justice levels.

## Competing interests

The authors declare that they have no competing interests.

## Authors' contributions

All authors read and approved the final manuscript. CBR and SSL were involved in data acquisition and statistical analysis and critically reviewed the manuscript. MM conceived the study, coordinated it, and drafted the manuscript.

## Pre-publication history

The pre-publication history for this paper can be accessed here:

http://www.biomedcentral.com/1471-244X/10/98/prepub
